# Development of an integrated fatigue measurement system for construction workers: a feasibility study

**DOI:** 10.1186/s12889-022-13973-5

**Published:** 2022-08-22

**Authors:** Sojeong Seong, Soyeon Park, Yong Han Ahn, Heejung Kim

**Affiliations:** 1 Department of Smart City Engineering, Hanyang University, 55 Hanyangdaehak-ro, Sangnok-gu, Ansan, Gyeonggi-do Republic of Korea; 2 School of Architecture and Architectural Engineering, Hanyang University, 55 Hanyangdaehak-ro, Sangnok-gu, Ansan, Gyeonggi-do Republic of Korea; 3 College of Nursing, Yonsei University, 50-1 Yonsei-ro, Seodaemun-gu, Seoul, Republic of Korea; 4 Mo-Im Kim Nursing Research Institute, Yonsei University, 50-1 Yonsei-ro, Seodaemun-gu, Seoul, Republic of Korea

**Keywords:** Fatigue, Construction worker, Ecological momentary assessment, Smartwatch, Feasibility study

## Abstract

**Background:**

Construction workers working in physically and mentally challenging environments experience high levels of occupational fatigue, which is the primary cause of industrial accidents and illnesses. Therefore, it is very important to measure fatigue in real time to manage the safety and health of construction workers. This study presents a novel approach for simultaneously measuring the subjective and objective fatigue of construction workers using ecological momentary assessment (EMA) and smartwatches. Due to the complexity and diversity of construction site environments, it is necessary to examine whether data collection using smartwatches is suitable in actual construction sites. This study aims to examine the feasibility of the integrated fatigue measurement method.

**Methods:**

This study comprised two phases: (1) development of an integrated fatigue measurement system for construction workers, and (2) a validation study to evaluate the method’s feasibility based on sensor data acquisition, EMA compliance, and feedback from construction workers in the field (*N* = 80). Three days of biometric data were collected through sensors embedded in the smartwatches for objective fatigue measurement, including heart rate, accelerometer, and gyroscope data. Two types of self-reported data regarding each worker’s fatigue were collected through a researcher-developed EMA application. The acceptability and usability of this system were examined based on the researchers’ observations and unstructured interviews.

**Results:**

Based on the standardized self-report questionnaire scores, participants were classified into high (*n* = 35, 43.75%) and low (*n* = 45, 56.25%) fatigue groups for comparison. The quantitative outcomes did not show a statistically significant difference between the two fatigue groups. Both groups experienced positive emotions and were able to recognize their health condition at the time of self-reporting, but stated that responding to this measurement system could be burdensome.

**Conclusions:**

This feasibility study provides a unique understanding of the applications of EMA and smartwatches for safety management in the construction workforce. The developed measurement system shows potential for monitoring fatigue based on the real-time collection of relevant data. It is expected that by expanding this integrated system through further research and onsite application, the health and safety of construction workers can be improved.

## Background

Construction workers experience high levels of occupational fatigue because they work in physically and mentally challenging environments. Occupational fatigue is the primary cause of industrial accidents due to increased work errors and reduced awareness of dangerous situations [1–3]. In 2019, the number of deaths in the private construction industry increased to 1061, the highest since 2007 [4]. In the United States, more than 200,000 cases of injuries and illnesses were reported among construction workers in 2019, resulting in a total of 79,700 days of labor loss [5]. Therefore, it is necessary to decrease the occupational fatigue of construction workers to ensure improved safety and health at construction sites.

Fatigue is defined as a loss of efficiency and lassitude or exhaustion resulting from bodily labor or mental exertion that inhibits motivation for any type of effort [6, 7]. Considering the complex and multidimensional nature of fatigue, there is ongoing research on measuring fatigue. Researchers have often focused on the causes and effects of fatigue as substitutes for fatigue itself [7, 8]. Previous studies identified factors that cause fatigue, for example, sleep deprivation, long work shifts, and demanding physical or mental activities [3, 7]. Several researchers reported a reduction in safe working behavior, job performance, productivity, teamwork, and morale as a result of excessive fatigue [1, 7].

Many studies have traditionally used self-reported questionnaires to measure occupational fatigue [9–12]. The subjective measurement of fatigue is based on the perception of symptoms that people experience when they feel physically, mentally, and functionally exhausted [8]. When subjectively measuring fatigue, evaluating self-reported symptoms of mental and physical fatigue is typical [8, 13, 14]. However, certain questionnaires suffer from methodological limitations in the assessment of objective and real-time fatigue, because most of these measures require recalling the past few days, weeks, or months [15]. Several studies suggest that it is necessary to measure real-time fatigue multiple times in a day to consider the characteristics of rapid changes in fatigue [8, 16]. Ecological momentary assessment (EMA) could be applicable in this regard.

EMA is defined as a subjective and repetitive evaluation of time-varying variables in real time under natural settings [17]; it is a method of repeatedly collecting the data reported by subjects in real time for individual symptoms, affects, and behaviors in a natural environment [18]. It allows research participants to report emotions, thoughts, and behaviors experienced through portable electronic devices (such as smartphones, actigraphy, and personal digital assistants) that they can carry in their daily lives [19–21]. Compared with retrospective self-reports, EMA is advantageous in that it decreases the subject’s recall bias and can also evaluate temporary associations among several variables simultaneously [17, 18]. It provides information pertaining to the contextual changes in subjects [18, 22]. Recently, EMA has been applied in several health studies to understand depression, addiction, and general fatigue [17, 20, 21, 23–25]. However, no attempt has been made yet to use this method to assess occupational fatigue of construction workers.

Researchers from diverse fields have developed fatigue instruments using new technologies. Objectively observable variables include neuronal activity or cardiorespiratory metrics [26, 27]. Objective fatigue measurements detect physiological indicators (for example, skin temperature, electroencephalogram, heart rate, muscle fatigue, eyelid movement, and energy consumption of physical activities), biometric indicators (for example, posture change, jerk, and head nodding), and cognitive fatigue indicators (for example, reaction time) [8, 15]. Although this approach provides real-time objective proxy data, it fails to capture subjective data. Thus, it is necessary to integrate both subjective and objective measures simultaneously to comprehensively capture real time data with respect to the natural environment [13, 16, 28].

Several studies have focused on testing wearable sensors for the objective measurement of the fatigue of construction workers [26, 27, 29]. These studies used physiological metrics such as heart rate, surface electromyography, and skin temperature [2, 26, 27, 29–31]. Previous studies quantified physical fatigue [26, 31] and verified the accuracy and reliability of wearable sensing technology to measure the physical fatigue of construction workers in real time through physiological metrics [27, 29]. However, the majority of these studies were conducted with a relatively limited sample size in a controlled laboratory environment, rather than in the real world and natural environment [2, 27, 30, 32]. Due to the complex and diverse working environment of a construction site, it is necessary to examine whether data collection using wearable sensors is suitable at actual construction sites. Thus, this study aims to examine the feasibility of both subjective and objective fatigue measurements using wearable devices for construction workers. We (1) developed an integrated system to measure the fatigue of construction workers using both subjective and objective data; (2) examined the feasibility and usability of applying this newly developed system for construction workers in the field; and (3) discussed the contextual and methodological challenges during the implementation of this system.

## Methods

This study comprises two phases: (1) development of an integrated fatigue measurement system for construction workers using both subjective and objective real-time data and (2) evaluation of feasibility and usability based on the feedback of construction workers in the field.

### Phase 1: development of an integrated fatigue measurement system

**Fig. 1:**
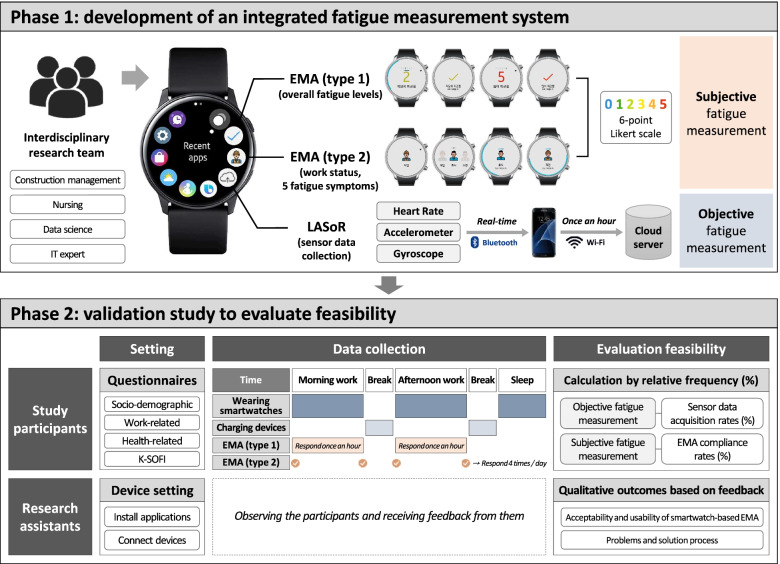
Integrated fatigue measurement system procedure

This measurement system includes a smartwatch (Galaxy Watch Active 2; Samsung Electronics Co., Ltd., Republic of Korea), an application for sensor data collection, LASoR (LK2 Consulting, Republic of Korea), and the developed EMA application as well as a smartphone (Galaxy S7 or later released; Samsung Electronics Co., Ltd., Republic of Korea). Our interdisciplinary research team developed the integrated fatigue measure using both wearable devices and the EMA methodology. The interdisciplinary research team comprised construction management, nursing, data science, and IT experts for sensor data collection. We conducted a preliminary survey on 108 construction workers and selected a smartwatch as the wearable device. We also consulted user interface designers who confirmed that the user interface design of the EMA application was better supplemented by inserting emoticons, such as by applying a color scale. The collected EMA data were stored in local storage. In addition, the smartwatch sent the collected sensor data to the smartphone via Bluetooth in real time, and the smartphone sent the data to the cloud server via Wi-Fi every hour (Fig. 1).

#### Smartwatch and smartphone

The main hub of our system is a smartwatch (Galaxy Watch Active 2; Samsung Electronics Co., Ltd., Republic of Korea) and smartphone (Galaxy S7 or later released; Samsung Electronics Co., Ltd., Republic of Korea). The product dimensions of the smartwatch are 1.6 × 1.6 × 0.41 in., and its weight is 26 g. It has an internal storage of 1.4 GB and a battery life of up to 95 h per charge. This smartwatch was selected due to its several advantages. Being based on the Korean language, it has an easy-to-understand interface compared with other products. In addition, it is also more user friendly because it provides various applications and functions that are well-integrated in the Android OS that is mostly used by Korean construction workers. It also uses Wi-Fi and is efficient at sending data.

#### Application for sensor data collection, LASoR

This smartwatch consists of several passive sensors including an accelerometer, gyro sensor, heart rate sensor, light sensor, and global positioning system. It can collect various biometric signals, including physical activity, sleep patterns, and psychological distress. The biometric data collected through sensors embedded in the smartwatch include heart rate, three-axis accelerometer, and three-axis gyroscope data. Heart rate is the most widely used form of physiological information for personal health status [2, 33]. Photoplethysmography (PPG) sensors are used to measure heart rate. Accelerometer and gyroscope data are collected to evaluate the amount of activity of workers. In previous studies, the feasibility of recognizing the activity of workers was verified using smartwatch acceleration data, without interfering with their ongoing work [34, 35]. The three-axis acceleration is the acceleration force data along each axis (x, y, and z axes) collected from the accelerometer, and the three-axis gyroscope is the rotational speed around each axis. These data can be used to detect motion and measure the amount of activity. The three-axis accelerometer, which measures inertial body motions, provides information-rich data regarding the workers’ activities, without considerable additional computational expenses [36].

#### EMA application

We developed the EMA application to load on the smartwatch. Our interdisciplinary research team consulted construction site managers and occupational nurses to detect symptoms of fatigue in a timely manner without interfering with the daily lives of the construction workers. We have developed applications for smartwatches that can respond to questions, considering that it is difficult for construction workers to use their smartphones to respond while working. Because construction workers wear protective gloves during work, the application was developed such that they can respond even when wearing gloves by pressing a button on the smartwatch.

The EMA app collected two types of self-reported data regarding the worker’s fatigue. First, the worker reports the overall fatigue levels after receiving an hourly alarm prompt (hereinafter referred to as EMA (type 1)). The worker answers the second EMA question regarding the participant’s fatigue symptoms four times a day when (a) starting the work, (b) taking regular breaks, and (c) finishing the work (hereinafter referred to as EMA (type 2)). Five specific questions were extracted from the Korean version of the Swedish occupational fatigue inventory (K-SOFI) [16] to evaluate momentary occupational fatigue among construction workers. In this study, the fatigue level was measured on a 6-point Likert scale (0 = “not at all”, 5 = “severe fatigue”). Originally, the SOFI was on a 7-point Likert scale (0 = “not at all”, 6 = “very high level”) [9]; however, we revised it to a 6-point Likert scale based on the findings from interviews with construction site managers and several construction workers that high levels of fatigue were not frequent [16].

### Phase 2: validation study to evaluate feasibility

In the second phase, we collected the data from 100 construction workers in the field and evaluated data acquisition, compliance with EMA, feasibility, acceptability, and usability. Feasibility of this developed system was assessed in line with recommendations of previous studies [18, 37, 38] using the following metrics: (a) sensor data acquisition rates as an objective fatigue measurement, (b) rates of EMA compliance as subjective fatigue measurement, and (c) self-reported acceptability and usability of smartwatch-based EMA. All study participants provided informed consent, and the study design was approved by the Institutional Review Board of the affiliated university (IRB No. XXX-2019-11-001 for anonymous review). Researchers explained the purpose, protocol, and strategies to the construction site manager working on site to protect personal information.

#### Study participants

Because this feasibility study measured fatigue by using EMA and collecting physiological data of construction workers, the sample size was determined by referring to previous studies. First, according to studies related to physiological data collection of construction workers, the advantage of sensor data measurement is that it can be analyzed with relatively few subjects (10–25 people) compared to self-report studies such as surveys [2, 26, 27, 29]. Second, in previous studies on the EMA of fatigue, approximately 40–80 participants were analyzed [23, 39, 40]. The number of subjects to be used for analysis was chosen to be 80, referring to previous studies, and the sample size was calculated as 100 corresponding to the expected dropout rate of 20%.

**Fig. 2:**
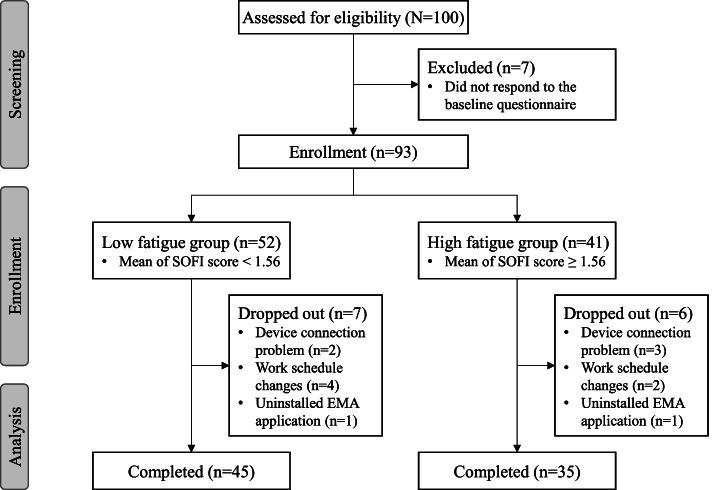
Participant flow diagram

A sample of 100 Korean construction workers was enrolled from five construction sites. Participants were recruited via an announcement posted on the bulletin boards or via word-of-mouth, and the construction site managers assisted recruiting the participants. The inclusion criteria were as follows: (1) age ≥ 19 years, (2) the ability to use a smartwatch and smartphone, and (3) the ability to understand the EMA instructions. The exclusion criteria were as follows: (1) non-Korean workers and (2) workers using a smartphone other than an Android smartphone. After checking the data completeness, seven participants were excluded from the analyses due to data for classifying fatigue groups. Of the 93 study participants, 80 completed the entire protocol: (a) five dropped out during data collection due to device connection problems, (b) six dropped out due to work schedule changes, and (c) two dropped out by accidentally uninstalling the EMA app during the study. The final data of 80 participants were included for the data analyses (Fig. 2).

#### Measures

Study participants completed the baseline questionnaires on the socio-demographic, work-related, and health-related characteristics. Socio-demographic characteristics included age, sex, marital status, education, and living status. Work-related characteristics include work experience, working hours of a day, employment form, and working intensity. Health-related characteristics include height, weight, smoking, drinking, and exercise.

K-SOFI was used to assess fatigue levels at the baseline for classification into the high and low fatigue groups [16]. The SOFI is internationally used to measure self-reported fatigue [9]. It comprises measures for lack of energy, physical exertion, physical discomfort, lack of motivation, and sleepiness (range: 0–120) [13]. The Korean version of the SOFI has demonstrated suitable reliability (Cronbach alpha ranging from .70 to .90) and has been tested on construction workers [16]. Based on the mean value of the K-SOFI of the participants, 1.56 (SD = 1.28), the high fatigue (higher than 1.56) and the low fatigue groups (lower than 1.56) were classified through a group comparison, based on the data acquisition rate or compliance with EMA.

#### Data collection procedure

The data were collected using standardized self-report questionnaires, K-SOFI, and the smartwatches with the EMA application were installed between July and November 2020. Our strategy was to evaluate construction workers’ fatigue over three working days using the developed system. The research team installed an application for data transfer on their smartphones. Trained research assistants explained the purpose of the self-reported EMA and smartwatches. In order to facilitate accurate self-reports by the participants and maintain strong inter-rater and intra-rater reliability, we provided video supplements to explain the approach to EMAs and the specific fatigue symptoms depending on levels. Whenever needed, trained research assistants additionally taught the participants to operate the smartwatches. Participants were expected to wear the smartwatch at all times, except during charging and bathing. To manage battery limitations, researchers instructed the participants to charge their smartwatches daily. To incentivize involvement, researchers encouraged the completion of all measures and indicated the remote monitoring of compliance; additionally, participants received a reward worth US $100 during the study.

## Results

### General characteristics of the study participant

Based on the mean value of the SOFI score, 35 participants were classified as the high fatigue group (43.75%) and 45 as the low fatigue group (56.25%). The majority of the men were middle-aged, who were married and living with their family. Their average work experience is approximately 11.81 ± 10.70 years. They work an average of 8 to 10 h each day. There was no difference between the high and low fatigue groups in terms of most of the general characteristics. However, the high fatigue group reported poorer levels of perceived health status (χ^2^ = 9.046, *p* < 0.05), and the low fatigue group reported longer sleep times the day before work (t = 1.786, *p* = 0.078; Table 1).

**Table 1:** Differences in general characteristics between the two fatigue groups (*N* = 80)

Variables	Total (***N*** = 80)	High fatigue group (***n*** = 35)	Low fatigue group (***n*** = 45)	***p***
**Age, mean (SD)**	44.86 (11.49)	46.71 (11.40)	43.42 (11.48)	.206
**Work experience (year), mean (SD)**	11.81 (10.70)	12.17 (9.98)	11.53 (11.34)	.793
**Sex, n (%)**				.207
Male	78 (97.5)	35 (100.0)	43 (95.56)	
Female	2 (2.5)	0 (0.0)	2 (4.44)	
**Marital status, n (%)**^**a**^				.547
Not married	26 (32.5)	10 (28.6)	16 (35.5)	
Married	48 (60.0)	23 (65.7)	25 (55.6)	
Divorced or separated	4 (5.0)	1 (2.85)	3 (6.7)	
**Education, n (%)**^**a**^				.701
Middle school	4 (5.0)	1 (2.9)	3 (6.7)	
High school	34 (42.5)	16 (45.7)	18 (40.0)	
College or above	41 (51.2)	18 (51.4)	23 (51.1)	
**Living status, n (%)**^**a**^				.730
Living alone	12 (15.0)	5 (14.3)	7 (15.6)	
Living with family members	53 (66.3)	25 (71.4)	28 (62.2)	
Living with nonfamily members	14 (17.5)	5 (14.3)	9 (20.0)	
**Working hour of a day, hour (%)**				.750
Less than 8 h	24 (30.0)	9 (25.7)	15 (33.4)	
8 to 10 h	41 (51.2)	18 (51.4)	23 (51.1)	
10 to 12 h	12 (15.0)	6 (17.1)	6 (13.3)	
12 h or more	3 (3.8)	2 (5.7)	1 (2.2)	
**Employment form, n (%)**^**a**^				.130
Full-time	21 (26.25)	12 (34.3)	9 (20.0)	
Dispatched	6 (7.5)	1 (2.9)	5 (11.1)	
Contract	27 (33.75)	10 (28.6)	17 (37.8)	
Temporary	19 (23.8)	11 (31.4)	8 (17.8)	
Others	6 (7.5)	1 (2.8)	5 (11.1)	
**Work intensity, n (%)**				.299
Extremely hard	6 (7.5)	3 (8.6)	3 (6.7)	
Hard	31 (38.7)	17 (48.5)	14 (31.1)	
Normal	37 (46.3)	12 (34.3)	25 (55.6)	
Easy	5 (6.3)	3 (8.6)	2 (4.4)	
Extremely easy	1 (1.2)	0 (0.0)	1 (2.2)	
**Smoking, n (%)**				.946
Never smoker	24 (30.0)	10 (28.6)	14 (31.1)	
Current smoker	52 (65.0)	23 (65.7)	29 (64.4)	
Past smoker	4 (5.0)	2 (5.7)	2 (4.4)	
**Drinking, n (%)**				.873
No	22 (27.5)	8 (22.9)	14 (31.1)	
2–3 times a month	16 (20.0)	7 (20.0)	9 (20.0)	
1–2 times a week	23 (28.7)	12 (34.3)	11 (24.5)	
3–4 times a week	17 (21.3)	7 (20.0)	10 (22.2)	
Everyday	2 (2.5)	1 (2.8)	1 (2.2)	
**Exercise, n (%)**				.373
Almost every day	4 (5.0)	2 (5.7)	2 (4.4)	
3–4 times a week	8 (10.0)	3 (8.6)	5 (11.1)	
2–3 times a week	4 (5.0)	3 (8.6)	1 (2.2)	
1–2 times a week	31 (38.8)	10 (28.6)	21 (46.7)	
No	33 (41.2)	17 (48.5)	16 (35.6)	
**Perceived health status, n (%)**^**a**^				.029^*^
Good	15 (18.8)	3 (8.6)	12 (26.7)	
Moderate	51 (63.7)	24 (68.6)	27 (60.0)	
Poor	9 (11.3)	7 (20.0)	2 (4.4)	
**Sleep time the day before work, hour (SD)**	6.449 (1.19)	6.186 (1.01)	6.659 (1.28)	.078

^*^P-value<.05

^a^missing data included

### Quantitative outcomes

#### Sensor data acquisition rates

Three types of sensor data were collected using smartwatches: heart rate, three-axis accelerometer, and three-axis gyroscope data. Heart rate data is the average 1-min rate measured using a PPG sensor. The data of each of the 80 final participants were collected every second and aggregated into three time zones: morning work, afternoon work, and sleep time. Valid data were sensor data measured from participants wearing smartwatches during work or sleep, and a total of 9,632,061 HR, 10,840,297 three-axis accelerometer, and 10,840,297 three-axis gyroscope valid data were collected. Sensor data collected from smartwatches through the application were organized in chronological order on each participant’s Excel sheet. We calculated whether sensor data for each participant were collected without omission every second based on the work and sleep times recorded by the workers. The sensor data acquisition rate for each participant was defined as the ratio of valid data to total data. Table 2 shows a comparison of sensor data acquisition rates for each fatigue group across the three time zones. The sensor data acquisition rate for each time zone was calculated as follows: the sum of the three-day valid data counts for each time zone divided by the total number of data should be collected for each time zone for 3 days. The mean acquisition rate of accelerometer and gyroscope data is 86.55% (95% CI 82.39–90.18%). However, the mean heart rate data acquisition rate is 76.58% (95% CI 70.50–81.86%), which is lower than the mean acquisition rate of the accelerometer and gyroscope data. Among the high fatigue group, the average accelerometer and gyroscope data acquisition rate is 84.40% (95% CI 76.02–90.75%), while for the low fatigue group, the average is 88.23% (95% CI 83.67–92.28%). For heart rate, the average data acquisition rate among the high fatigue group is 75.16% (95% CI 64.58–83.92%) and for the low fatigue group, it is 77.67% (95% CI 70.36–84.18%). There was no statistically significant correlation between the two fatigue groups across all the three types of sensor data.

**Table 2:** Sensor data acquisition rate by type per group

Variable	Total (***N*** = 80) Mean (95% CI)	High fatigue group (***n*** = 35) Mean (95% CI)	Low fatigue group (***n*** = 45) Mean (95% CI)
**Data acquisition rate (%) during morning work**			
Heart rate	79.87% (73.59–85.17%)	77.85% (67.26–86.67%)	81.44% (73.81–88.34%)
Accelerometer	92.75% (88.85–96.39%)	91.70% (83.15–97.61%)	93.57% (89.21–97.28%)
Gyroscope	92.75% (88.85–96.39%)	91.70% (83.15–97.61%)	93.57% (89.21–97.28%)
**Data acquisition rate (%) during afternoon work**			
Heart rate	79.13% (72.48–84.68%)	79.68% (67.67–89.85%)	78.71% (70.52–85.92%)
Accelerometer	91.41% (87.20–94.90%)	91.68% (84.01–97.94%)	91.20% (86.38–95.64%)
Gyroscope	91.41% (87.20–94.90%)	91.68% (84.01–97.94%)	91.20% (86.38–95.64%)
**Data acquisition rate (%) during sleep time**			
Heart rate	67.81% (59.99–75.88%)	64.31% (49.14–76.92%)	70.52% (59.15–81.64%)
Accelerometer	72.54% (64.68–80.17%)	65.92% (50.68–78.72%)	77.69% (68.20–86.63%)
Gyroscope	72.54% (64.68–80.17%)	65.92% (50.68–78.72%)	77.69% (68.20–86.63%)

#### EMA compliance rates

In a three-day experiment, two types of EMA data were collected using the app loaded on the smartwatches. For EMA (type 1), comprising a simple questionnaire on subjective individual fatigue on the 6-point Likert scale, the maximum amount of data was 2469 during the 3-day study period. It was calculated as the number of times each participant responded per hour within the actual working hours. A total of 1910 (77.36%) EMA (type 1) data were collected, 1767 (71.57%) of which were valid for analysis. Some participants responded again within minutes to this value, although ideally, responses were only expected once an hour. These values were treated as follows: (a) if re-entered within 5 min of the first value, the last value was considered as valid data; and (b) if re-entered 5 min after the first value, the average of all the values was considered to be the valid data. Of the 80 participants, 65 (81.25%) recorded valid data for EMA (type 1) at least 6 times a day on average. The EMA compliance rate was calculated as the ratio of valid data to the maximum amount of data for each participant. The compliance rate of EMA (type 1) among the high fatigue group was 71.53% (95% CI 64.94–77.35%), and the compliance rate of EMA (type 1) among the low fatigue group was 71.52% (95% CI 66.07–76.69%); thus, there was no significant difference between the two fatigue groups.

For EMA (type 2), consisting of five questions that measure momentary symptoms of fatigue, the maximum amount of data planned was 960. It was calculated by multiplying the number of participants (80 samples) with the participation period (3 days) and the number of changes in the work state (4 times per day). A total of 735 (76.56%) EMA (type 2) data were collected, 618 (64.38%) of which were valid for analysis. The compliance rate of EMA (type 2) among the high fatigue group was 66.19% (95% CI 55.91–75.62%), and the compliance rate of EMA (type 2) among the low fatigue group was 62.96% (95% CI 53.60–70.66%); thus, there was no significant difference between the two fatigue groups.

The difference in the two types of EMA compliance rates between the fatigue groups was not statistically significant, but the average compliance rate of the high fatigue group was slightly higher in both EMA responses. However, when analyzing the correlation between the general characteristics of the participant and the compliance rates through further analysis, EMA (type 2) showed a significant correlation with age (r = −.287, *p* = 0.01), but EMA (type 1) did not exhibit any statistically significant correlation.

### Qualitative outcomes

#### Acceptability and usability

The acceptability and usability of this system were examined based on the observations and unstructured interviews performed by the research team. Both groups felt positive emotions while using this fatigue measurement system. Based on open-ended feedback, participants expressed perceptions of acceptability and usability through representative quotes such as “assisted in recognizing fatigue at the time of self-reporting” and “allowed me to ponder upon my health condition.” However, there were also negative perceptions, reflecting dissatisfaction or discomfort while using this system. Representative quotes for this perception included, “it was uncomfortable to wear it because I do not usually wear a watch,” and “sometimes I felt burdened owing to interference with work.” Participants also cited the burden of responding as reasons for EMA non-compliance owing to difficulty in recognizing the individual fatigue level experienced.

#### Problems and solution

During the study, a few problems were encountered. Device errors sometimes occurred while using the smartwatch. For example, when sensor data stored in the smartwatch were not transmitted to the smartphone in real time, they had to be transmitted manually by pressing the transfer button; the main reasons for this were that the participants had accidentally turned off Bluetooth or experienced interruptions in Internet connectivity. In addition, for a worker located underground, the data transmission was delayed or data loss occurred due to network communication problems. Furthermore, the smartwatches were sometimes discharged while working, despite regular charging by the participants. Due to the nature of work of construction workers, they often worked from early morning to late evening, which hindered securing the charging time. Because sleep was important for alleviating fatigue, we asked the participants to wear smartwatches during sleep time to collect physiological data during sleep that resulted in insufficient charging time. Therefore, this problem could be alleviated if construction workers wore smartwatches only during work and charged them at other times.

Participants were more aware of their condition and responded to them because EMA (type 2) was based on a questionnaire on five symptoms of fatigue. However, many of the participants said that they were significantly burdened because of the need to answer five questions, with their working state changing four times a day. Some participants mentioned that there were cases when they answered five questions without contemplating owing to time conflicts at the start of the work. We confirmed that most participants preferred to respond to EMA (type 1) over EMA (type 2).

## Discussion

This study aims to evaluate the feasibility of using an integrated system to measure fatigue of construction worker using EMA and smartwatches. We classified them into high and low fatigue groups based on SOFI scores and collected diverse types of data, such as the EMA to measure subjective fatigue while working, sensor data to measure objective fatigue, and self-report questionnaires. The high fatigue group reported poorer levels of perceived health status than the low fatigue group, but there was no significant difference between the two fatigue groups in terms of the other general characteristics. The quantitative outcomes showed that there was no significant difference in sensor data acquisition rates and EMA compliance rates between the two fatigue groups; however, the EMA (type 2) compliance rate was correlated with age. The qualitative outcomes showed that they experienced positive emotions by being interested in their health condition after using our fatigue measurement system. However, they also felt burdened while using the smartwatch because it often interrupted their work while responding to the scheduled EMA.

In the case of sensor data, a relatively high acquisition rate was realized merely by wearing a smartwatch on the wrist. In this study, the mean working time of participants is 10 ± 1.18 h a day, and the mean time of the accelerometer and gyroscope data collection is 9.3 ± 2.08 h a day, accounting for 92.9%. The reasons for the missing data are as follows: (a) Internet connection was disconnected for a long time due to underground work or user manipulation error, (b) the participant did not wear the smartwatch because its battery was discharged or the participant forgot to wear the smartwatch, and (c) smartwatch or data-collection application errors. The mean of heart rate data acquisition rate was lower than the accelerometer and gyroscope data because there were missing values owing to lose attachment of smartwatch on the wrist. Most wristband-type wearable devices use PPG sensors for heart rate measurement, and several researchers have attempted to improve noise problems in heart rate data obtained from the daily exercises of subjects in controlled laboratory environments and to evaluate the accuracy of PPG-based heart rate monitoring [29, 41–43]. However, because PPG signals are sensitive to motion artifacts caused by user movement during data acquisition [29, 42], more missing values in heart rate than accelerometers and gyroscopes occurred due to signal noise, when data were collected from construction workers with considerable physical movements.

The findings of this study suggested that EMA is a feasible and useful methodology that promises potential applications in the construction industry. The method of fatigue measurement of construction worker using smartwatch with EMA can provide symptoms of fatigue subjectively and objectively in real time. It is possible to monitor the individual pattern and examine fatigue fluctuations during a day. The two types of self-reported EMA data demonstrated suitable compliance rates compared to other feasibility studies on EMA [37, 44, 45]. The use of EMA in the construction population is novel, and additional research is perhaps required to better understand and maximize EMA acceptability and compliance among construction workers suffering from occupational fatigue. EMA was originally conducted individually; however, the peer-support system proved effective owing to the collective working environment of construction workers in the current study. Despite the tendency of older workers to avoid using mobile technology, younger coworkers assisted in responding to their EMA. EMA (type 1) comprising a simple question was appropriately collected regardless of age; however, in the case of EMA (type 2) comprising five questions, the compliance rate was lower for older workers. Workers assisted one another in responding to EMA (type 1) because the prompt sounded at a set time during work, but in case of EMA (type 2) that necessitated response at a time when individual work status changed, the older workers unfamiliar with smartwatches faced difficulty responding. This suggests that effective strategies are required to enhance compliance and acceptability in this population. Thus, in case of EMA (Type 2), it is necessary that the safety or health officers at construction sites remind the older workers at the start or end of work. It is also important to select valid questions to measure momentary fatigue of construction workers. Therefore, researchers should consider its practicality, select the appropriate instrument, and train workers [17].

Through interviews with construction workers who participated in this study, we found that they were more interested in health than expected. Some participants reported faithfully responding to EMA because they wanted to check their health status. In previous studies, most monitoring systems for application in construction sites focused on allowing safety managers or site managers to check the workers’ conditions in real time [2, 27, 29, 46]. Researchers of previous studies indicated that early detection of fatigue symptoms of construction workers in the field enables timely interventions such as rests [15]. Through this study, it is expected to enable voluntary and active safety and health management that motivates construction workers by facilitating the recording of their fatigue condition during work.

However, several methodological issues were identified in this study, which provide valuable information for the further development of fatigue measurement systems for construction workers. Sleep is an important factor that affects workers’ fatigue [47], but collecting sleep data using a smartwatch causes battery discharge problems. In this study, 27.5% of the participants did not wear smartwatches while sleeping, and they reported the inconvenience of wearing a smartwatch while sleeping, followed by the lack of battery charging time. Thus, the collection of sleep data using smartwatches is not feasible, and this should be modified in future studies. In addition, one of the important factors to consider when applying wearable sensing technology to the industry is the social and privacy issues [15, 48]. A few workers expressed concern over their personal information being potentially stored on the device. Because wearable sensing technology is vulnerable to data security risks, strong security measures must be adopted to protect the workers’ personal information from security threats for actual construction site application [49].

Overall, while the study showed acceptance and feasibility of this developed system, there are several limitations in this study. First, we used an instrument that is internationally used to assess occupational fatigue for classifying the participants into two fatigue groups, because this instrument has shown suitable reliability and has been tested on Korean construction workers [16]. However, defining a fatigue group may require further evaluation based on assessment of fatigue caused by various complex factors. In addition, the participants were recruited at the construction site of large construction companies in Korea; therefore, they may not appropriately reflect the general construction population owing to cultural and institutional differences. Hence, it is necessary to address this issue by evaluating the feasibility of incorporating construction workers belonging to various countries and cultures in future research. Moreover, the use of incentives and researchers’ bias may have caused an overstatement of compliance.

## Conclusions

Our fatigue measurement system is a novel approach that integrates wearable sensing technology and EMA methodology and can be utilized for subjectively and objectively measuring the fatigue of construction workers. Many systems using wearable sensing technology have been developed to obtain only physiological data of construction workers. Moreover, the previous studies did not collect actual data from workers at construction sites and did not adopt EMA for subjective fatigue data collection. By contrast, our system was applied to actual construction sites and integrated self-reported data on fatigue into the system leveraging EMA methodology. To obtain both subjective and objective fatigue data, we implemented a cloud-based IoT system comprising a smartwatch, a smartphone, and an EMA application, made feasible by our interdisciplinary research team through collaboration with several experts working in the healthcare and ICT industry. We found that objective and subjective fatigue symptoms were appropriately collected by the proposed fatigue measurement system based on wearable sensing technology and EMA. The developed fatigue monitoring system can reduce the accident rates of construction workers and provide an opportunity to develop a fatigue management system. However, the current feasibility study is limited to describing only a proportion of data acquisition. Thus, future studies should further investigate detailing sensor data to develop future fatigue monitoring systems for the construction industry.

## Data Availability

The datasets generated and/or analyzed during the current study are not publicly available due to the informed consent form distributed to all subjects to inform them that the data collected in the study would not be provided to third parties who did not participate in the study, but are available from the corresponding author on reasonable request.

## Abbreviations

CI: Confidence interval
EMA: Ecological momentary assessment
ICT: Information and communication technology
IoT: Internet of things
IT: Information technology
PPG: Photoplethysmography
SOFI: Swedish occupational fatigue inventory
